# Combination therapy of infliximab and thalidomide for refractory entero-Behcet's disease: a case report

**DOI:** 10.1186/1471-230X-13-167

**Published:** 2013-12-09

**Authors:** Yue Li, Zelong Han, Xianfei Wang, Zhihui Mo, Wei Zhang, Aimin Li, Side Liu

**Affiliations:** 1 Guangdong Provincial Key Laboratory of Gastroenterology, Department of Gastroenterology, Nanfang Hospital, Southern Medical University, Guangzhou 510515, China; 2 Department of Gastroenterology, Affiliated Hospital of North Sichuan Medical College, Nanchong, China

**Keywords:** Entero-Behcet's disease, Infliximab, Thalidomide

## Abstract

**Background:**

Behcet's disease (BD) is a systemic inflammatory disease with the histopathological features of leukocytoclastic vasculitis that affects nearly all organs and systems. When it involves the intestine, it is called entero-Behcet's disease (entero-BD).

**Case presentation:**

Here we described a 23-year-old man with entero-BD refractory to conventional therapies who responded well to the combination therapy of infliximab, an anti-tumor-necrosis-factor (TNF)-alpha antibody, and thalidomide. After combination treatment, the patient’s symptoms improved greatly and his Crohn's Disease Activity Index (CDAI) score decreased from 344 to 52 points, accompanied by a body weight increase from 53 kg to 64 kg. A follow-up endoscopy performed 10 weeks after the treatment showed significant improvement and the patient's multiple ulcers had healed well.

**Conclusion:**

The combination therapy of infliximab and thalidomide appears to be clinically effective in a patient with refractory entero-BD. However, further studies need to be performed to evaluate the efficacy of this combination therapy.

## Background

Behcet's disease (BD) is a chronic relapsing vasculitis characterized by recurrent oral and genital ulcerations with uveitis and is more prevalent around countries along the ancient Silk Route [1]. Entero-Behcet's disease (entero-BD) is characterized by intestinal inflammation with round and oval ulcers typically in the ileocaecum and is associated with gastrointestinal symptoms, which are often uncontrollable, relapsing, and can cause acute intestinal bleeding or perforation [2,3]. Gastrointestinal involvement has been reported in 3%–26% of patients with BD [4]. The etiology of BD is still unknown, but tissue damage that occurs in BD patients is believed to be caused by oxygen radicals, which are generated by proinflammatory cytokines and arachidonic acid metabolites [5,6]. Although corticosteroids, 5-aminosalicylic acid derivatives, immunosuppressive agents, and immunomodulators have been used to treat BD patients with varying degrees of success, BD is associated with severe morbidity and considerable mortality [7]. Tumor necrosis factor (TNF)-alpha plays an important role in this T helper cell type 1 (Th1)-mediated disease [8]. Infliximab, a monoclonal antibody to TNF-alpha, which neutralizes TNF-alpha and down-regulates the expression of granulocyte-macrophage colony-stimulating factor has been demonstrated to be an effective therapy for Crohn's disease, rheumatoid arthritis and other Th1-mediated disorders [9]. However, the single use of infliximab is not efficient in all BD patients [10,11]. Thalidomide selectively inhibits the production of TNF-alpha in monocytes and reduces its activity by a mechanism distinct from infliximab [12,13]. Many publications have reported the possible use of thalidomide for a wide range of conditions such as BD [14-17]. Here, we utilized a combination therapy of infliximab and thalidomide and showed that it appears to be clinically effective in a patient with refractory entero-BD.

## Case presentation

**Figure 1:**
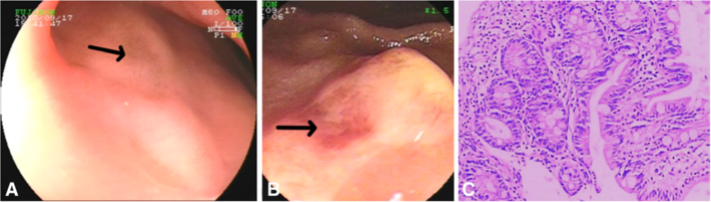
**Related auxiliary examinations were conducted in order to make a definitive diagnosis.** Gastroscopy revealed a scar (arrow) left on the anterior wall of the duodenum from a healed ulcer **(A)**. Double balloon enteroscopy revealed a round ulcer (arrow) characterized by hyperemia and erosion **(B)**. Biopsy specimens from the jejunum revealed chronic inflammatory infiltrate consisting of a mixture of neutrophils, lymphocytes and plasma cells **(C)**.

**Figure 2:**
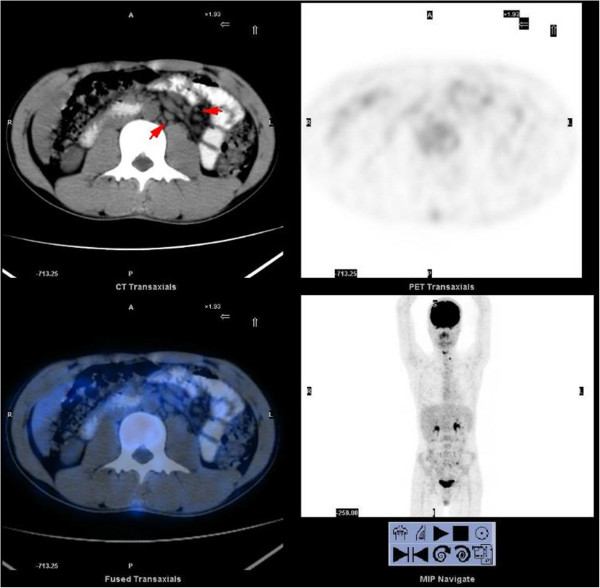
PET-CT revealed multiple enlarged lymph nodes (arrow) in the abdominal and mesenteric region without abnormal concentration of 18 F-fluorodeoxyglucose (18 F-FDG).

A 23-year-old man was admitted to our hospital because he had recurrent abdominal pain and fever for more than 2 years. The patient began to have a burning pain in the epigastrium in October 2008, which mostly occurred at night and when he was hungry. The pain occurred once every 1 to 2 months, each time lasting for 1 to 2 days, accompanied by fever, with temperature fluctuating between 38-39°C, which would alleviate by itself. The patient did not have diarrhea, night sweats or other symptoms. Laboratory examination in the local hospital revealed white blood cell (WBC) 10.0 × 10^9^/L (normal 3.6-9.7 × 10^9^/L), neutrophil rate 79.2% (normal 50–70%), hemoglobin 116 g/L (normal 120–160 g/L) and C-reactive protein (CRP) 87.7 mg/L (normal 0–5 mg/L). Erythrocyte sedimentation rate (ESR) was 27.0 mm/h (normal 0–15 mm/h), and occult blood test (OBT) was positive. The patient was a non-smoker with no family history of inflammatory bowel disease. Gastroscopy revealed duodenal bulb ulcers. Although acid inhibitors and antipyretics were used in the local hospital, his symptoms did not improve. He was referred to our department for further evaluation. On admission, a physical examination found an enlarged submental lymph node which was soft and removable without pressing pain. After admission, laboratory examination indicated that WBC, OB, CRP, and ESR were normal. The patient tested negative for autoantibodies to nuclear antigen, double-stranded deoxyribonucleic acid, nuclear ribonucleoprotein, anti-saccharomces cerevisiae antibodies and anti-neutrophil cytoplasmic antibodies. In addition, Pathergy and Widal tests were both negative. Gastroscopy and double balloon enteroscopy revealed duodenal bulb ulcers and scattered round small ulcers in the jejunum with no evidence of Helicobacter Pylori infection (Figure 1A, 1B). Biopsy of a deeply ulcerated area of jejunum revealed nonspecific mucosal inflammation without granulomata (Figure 1C). Positron emission tomography/computed tomography (PET-CT) found multiple and flake concentration around the jejunum and ileum. Inflammatory and hyperplastic lymph nodes without increased metabolism were found in the abdominal, retroperitoneal and mesenteric region (Figure 2).

**Figure 3:**
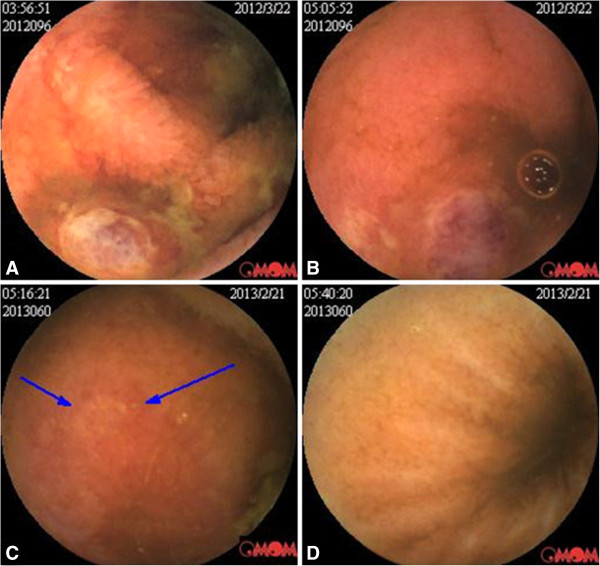
**Before infusion, capsule endoscopy showed multiple oval ulcers with purplish red color change and thin fur attached on the surface.** Congestion and edema of the mucosa can be seen around the ulcers which were widely distributed in the small intestine **(A, B)**. Repeated capsule endoscopy showed improvement in ulcerations and inflammation 10 weeks after the last infusion **(C, D)**. Most of the ulcers had healed, leaving a small sheet of erosion (arrow).

**Figure 4:**
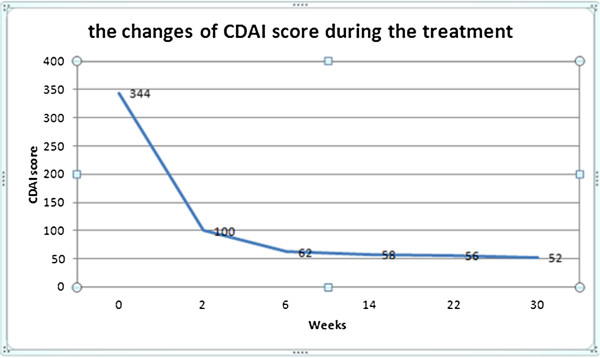
**A CDAI score <150 points is considered clinical remission in a patient with CD.** After the first infusion, the patient noticed great improvement in symptoms, and his CDAI score declined dramatically from 344 to 100 points, which indicated clinical remission.

Although various test had been conducted, the diagnosis of the patient remained unresolved. His medical history was long and he did not complaint of hematochezia. PET-CT scans did not reveal the presence of any abdominal mass nor any sign of neoplasma. Most significantly, the biopsy specimen from the deeply ulcerated area of the jejunum did not show the presence of lymphoma cells, therefore the possibility of intestinal lymphoma was excluded. Coeliac disease is more prevalent in Caucasian, but our patient is an Asian. Meanwhile, eating wheat foods does not have obvious relation with the patient’s symptoms. Therefore, the possibility of coeliac disease was also excluded. However Crohn's disease, tuberculosis of the intestines and BD were difficult to differentiate from each other and remained possible causative conditions. After discharge, the patient still suffered from recurrent abdominal pain. He returned to our department with recurrent abdominal pain and fever in March 2012. On further investigation it was discovered that the patient had had recurrent oral ulcers, genital ulcers and occasional blurred vision since childhood. Capsule endoscopy was carried out and revealed multiple ulcers in the small intestine, mainly located in the jejunum (Figure 3A, 3B). BD is diagnosed based on clinical evaluation. Our patient was diagnosed with entero-BD based on the International Criteria for Behcet’s Disease [18]. However, although he had been treated with prednisone, mesalazine, cyclophosphamide and colchicines in the local hospital, his symptoms had not improved. After discussion with the patient, a combination therapy including infliximab (5 mg/kg), thalidomide (100 mg,qd) and prednisone (15 mg/day) was prescribed. Infliximab was infused at weeks 0, 2, 6, 14, 22 and 30 according to instructions. No medication-related adverse reactions were observed and the prednisone dose was gradually tapered during the treatment. After the first infusion with infliximab the patient’s abdominal pain and fever disappeared despite complete withdrawal of steroids. CRP decreased from 87.7 mg/L to 0.7 mg/L and ESR decreased from 27.0 mm/h to 4 mm/h. Because most of his symptoms were gastrointestinal, the Crohn's Disease Activity Index (CDAI) was selected as an objective measure of response to the therapy. The CDAI score decreased from 344 to 52 points during therapeutic period (Figure 4). Meanwhile, his body weight increased from 53 kg to 64 kg. Capsule endoscopy performed 10 weeks after the last infusion showed marked endoscopic improvement and the patient's multiple ulcers had healed well (Figure 3C, 3D).

## Discussion

Although oral ulceration, genital ulceration and eye disease are classical triad symptoms of BD, the cardiovascular, gastrointestinal, musculoskeletal and central nervous systems can also be affected [19]. The pathogenesis of BD remains unknown but major determinants involving genetic and immune system abnormalities have been recently reported [20]. The diagnosis of entero-BD is made according to the International Criteria for Behcet’s Disease taking into account the patient’s gastrointestinal symptoms and intestinal ulcers as detected by endoscopy. However, when the patient’s symptoms are not typical, the diagnosis may be difficult. In intestinal BD, deep ulcers develop in the gastrointestinal tract, typically in the ileocaecum. However, the patient presented in this report had scattered small ulcers which were mainly in the jejunum. In addition, the pathergy test was negative, which also complicated the diagnosis. After collecting a detailed medical history, the diagnosis was eventually established. In our experience, comprehensive inquiry and a detailed medical history is important in the diagnosis of entero-BD as well as endoscopic examination. When gastroscopic and colonoscopic investigations do not reveal significant pathological changes, double-balloon enteroscopy or capsule endoscopy may contribute to the detection of lesions since many entero-BD ulcers are atypically located in the ileum and jejunum. Additionally, the pathergy test is not always positive and the clinician should not be confused by the negative results when making a diagnosis of BD.

Entero-BD is often difficult to manage by conventional therapies such as corticosteroids, 5-aminosalicylic acid derivatives and immunosuppressive agents. Recently, off-label use of anti-tumor necrosis factor agents for BD has increased, suggesting that TNF blockade represents an important therapeutic approach for patients with severe and resistant BD, but randomized controlled trials are lacking [7,21-23]. Infliximab has been reported to have rapid and excellent efficacy in patients with refractory entero-BD [24-27]. Although 91% of BD patients responded to infliximab, some patients responded poorly to the treatment of infliximab [10,11,21]. In our experience, entero-BD patients usually respond poorly to various conventional treatments such as prednisone, mesalazine, cyclophosphamide and colchicines, which were all used for the treatment of our patient in the local hospital. Since our patient failed to respond to various conventional treatments, along with the concern that he may respond poorly to a single use of infliximab, we decided to treat him with a combination therapy of infliximab and thalidomide, which may synergize due to a complete blockade of TNF-α.

After the treatment, his symptoms and intestinal lesions improved without adverse effects. To our knowledge, this is the first report of a treatment regime using the combination therapy of infliximab and thalidomide. The good response of the patient to this combination therapy is likely a result of a complete TNF-α blockade by the two agents. Although our report describes only one case, the improvement in symptoms of the patient supports evidence that increased levels of TNF-α play a critical role in the inflammatory process associated with BD. In view of limitations of the present treatment for intestinal BD, combination therapy with infliximab and thalidomide appears to be an effective approach for the treatment of entero-BD and perhaps other manifestations of BD.

## Conclusion

Based on our report, the combination therapy of infliximab and thalidomide could be selected as an effective approach for the patients with refractory entero-BD. However, further studies need to be performed to evaluate the efficacy of this combination therapy.

## Consent statement

Written informed consent was obtained from the patient for publication of this case report and any accompanying images. A copy of the written consent is available for review by the Editor of this journal.

## Abbreviations

BD: Behcet's disease; TNF: Tumor necrosis factor; WBC: White blood cell; CRP: C-reactive protein; ESR: Erythrocyte sedimentation rate; OB: Occult blood test; PET-CT: Positron emission tomography/computed tomography; CDAI: The Crohn's Disease Activity Index.

## Competing interests

We declare that we have no competing interests.

## Authors’ contributions

YL and ZH contributed equally to this paper. YL designed and drafted the manuscript. ZH participated in the design of the study. ZM and XW performed the data collecting of this case. WZ and AL helped to revise the manuscript. SL conceived of the study, and participated in its design and coordination and helped to draft the manuscript. All authors read and approved the final manuscript.

## Pre-publication history

The pre-publication history for this paper can be accessed here:

http://www.biomedcentral.com/1471-230X/13/167/prepub
